# Fixation of split-thickness skin graft using fast-clotting fibrin glue containing undiluted high-concentration thrombin or sutures: a comparison study

**DOI:** 10.1186/s40064-016-3599-x

**Published:** 2016-11-02

**Authors:** Hyun Ho Han, Daiwon Jun, Suk-Ho Moon, In Sook Kang, Min Cheol Kim

**Affiliations:** 1 Department of Plastic and Reconstructive Surgery, Incheon St. Mary’s Hospital, College of Medicine, The Catholic University of Korea, Seoul, Republic of Korea; 2 Department of Plastic and Reconstructive Surgery, Seoul St. Mary’s Hospital, College of Medicine, The Catholic University of Korea, Seoul, Republic of Korea; 3 Department of Plastic and Reconstructive Surgery,St. Vincent’s Hospital, College of Medicine, The Catholic University of Korea, Seoul, Republic of Korea

**Keywords:** Fibrin sealant, Suture, Thrombin, Fast clotting, Split-thickness skin graft

## Abstract

**Objective:**

For skin defects caused by full-thickness burns, trauma, or tumor tissue excision, skin grafting is one of the most convenient and useful treatment methods. In this situation, graft fixation is important in skin grafting. This study was performed to compare the effectiveness of skin graft fixation between high-concentration fibrin sealant and sutures. There have been numerous studies using fibrin sealant for graft fixation, but they utilized slow-clotting fibrin sealant containing less than 10 IU/mL thrombin.

**Method:**

Twenty-five patients underwent split-thickness skin grafting using fast-clotting fibrin sealant containing 400 IU/mL thrombin, while 30 patients underwent grafting using sutures. Rates of hematoma/seroma formation, graft dislocation, graft necrosis, and graft take were investigated postoperatively. The graft surface area was calculated using Image J software (National Institutes of Health, Bethesda, MD, USA).

**Result:**

After 5 days, rates of hematoma/seroma formation and graft dislocation were 7.84 and 1.29% in group I, and 9.55 and 1.45% in group II, respectively. After 30 days, rates of graft necrosis and graft take were 1.86 and 98.14% in group I, and 4.65 and 95.35% in group II. Undiluted fibrin sealant showed significantly superior results for all rates (*p* < 0.05) except graft dislocation.

**Conclusion:**

When high-concentration fast-clotting fibrin sealant was applied to skin grafts without dilution, no difficulty was experienced during surgery. Sealant showed superior results compared with sutures and had an excellent graft take rate.

**Level of evidence:**

II.

## Background

Skin grafting is one of the most convenient and useful treatment methods for skin defects caused by full-thickness burns, trauma, or tumor tissue excision. In skin grafting, graft fixation is important. Use of sutures or staples is a standard method for skin fixation (Zederfeldt 1994; Waiker and Shinalingappa 2015; Butts et al. 2015); however, the use of fibrin glue has also been suggested. Use of fibrin as an adhesive was introduced by Bergel in 1909, and Tidrick and Warner used fibrin for skin graft fixation for the first time in 1944 (Saltz et al. 1991). Since the 2000s, many instances of graft fixation using fibrin have been reported, but the process of obtaining fibrin remains difficult. Fibrin sealants prepared using autologous blood and ready-made sealants contain a low thrombin concentration of 5 IU/mL, which are restrictedly obtainable in Europe and Canada. Fibrin sealant products available in South Korea and the United States are fast-clotting, with a high thrombin concentration of 400 IU/mL; thus, they must be diluted to a lower concentration, which requires significant time and effort (Gibran et al. 2007; Mittermayr et al. 2006; Buckley et al. 1999; Currie et al. 2001). Fibrin containing 400 IU/mL thrombin is typically used for hemostasis. When used to facilitate skin grafting, however, clotting occurs before the split-thickness graft can be manipulated due to its short coagulation time. Accordingly, it is difficult to insert the graft into the wound bed.

There have been numerous studies using fibrin sealant for graft fixation, but they utilized slow-clotting fibrin sealant containing less than 10 IU/mL thrombin. In an attempt to eliminate the time and difficulty in diluting the thrombin concentration, in this study, a fast-clotting fibrin sealant containing a high thrombin concentration without dilution was applied to the skin for graft fixation. Then, we compared the results of graft fixation using silk suturesor staples.

## Methods

Analysis of split-thickness skin grafting for skin defects of upper and lower extremities in 55 patients at Seoul St. Mary’s Hospital was conducted between March 2009 and December 2012. The study design was reviewed and accepted by the Institutional Review Board (The registration number: KC14RISE0914).

**Fig. 1:**
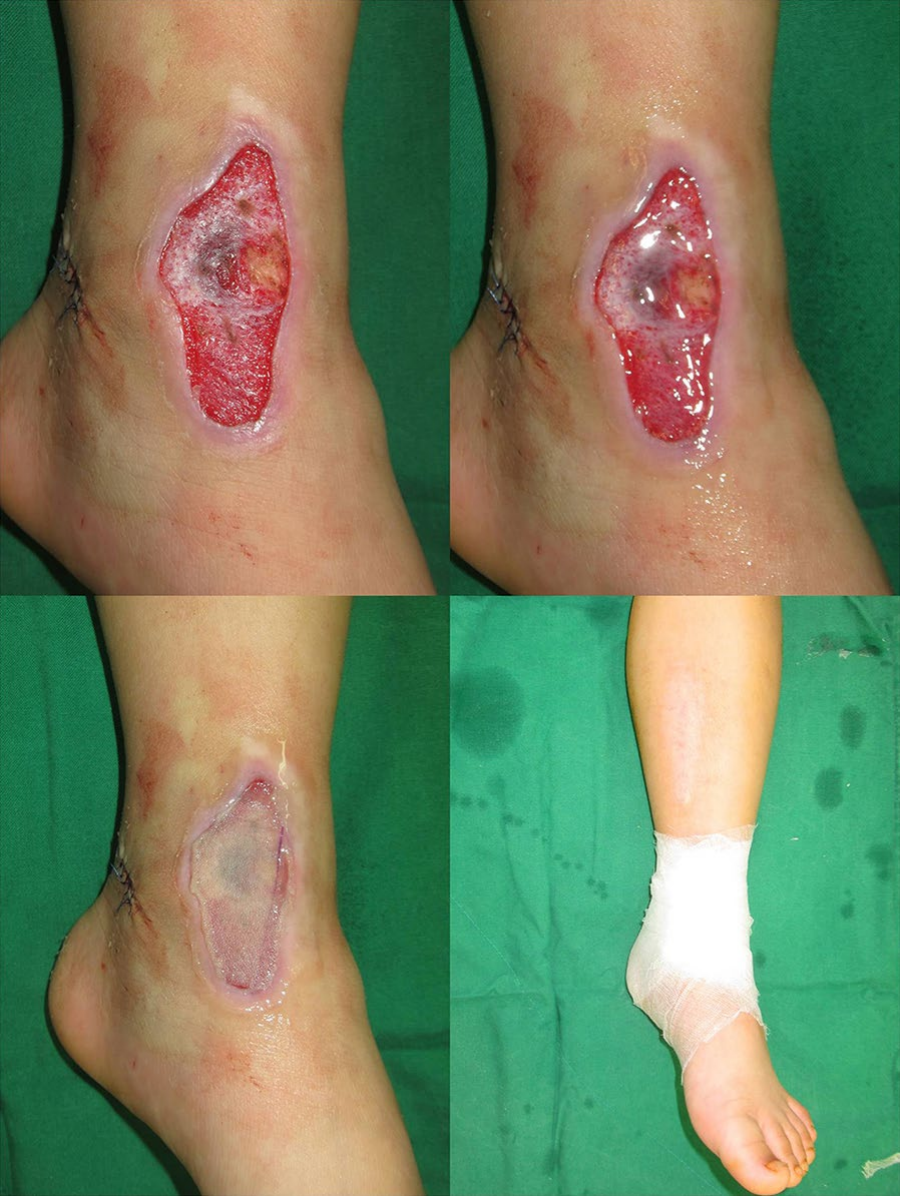
(*left above*) Leftankle skin defect due to a pedestrian accident in a 6-year-old boy. (*right above*) Fast-clotting fibrin sealant containing 400 IU/mL thrombinwas sprayed on the healthy wound bed with a thickness of 0.05 mL/cm^2^. Fibrin clotting was already observed before applying the split-thickness skin graft. (*left below*) Fibrin sealant was applied once more to the graft boundary. (*right below*) Moderate compressive dressing was performed using a gauze bandage

**Fig. 2:**
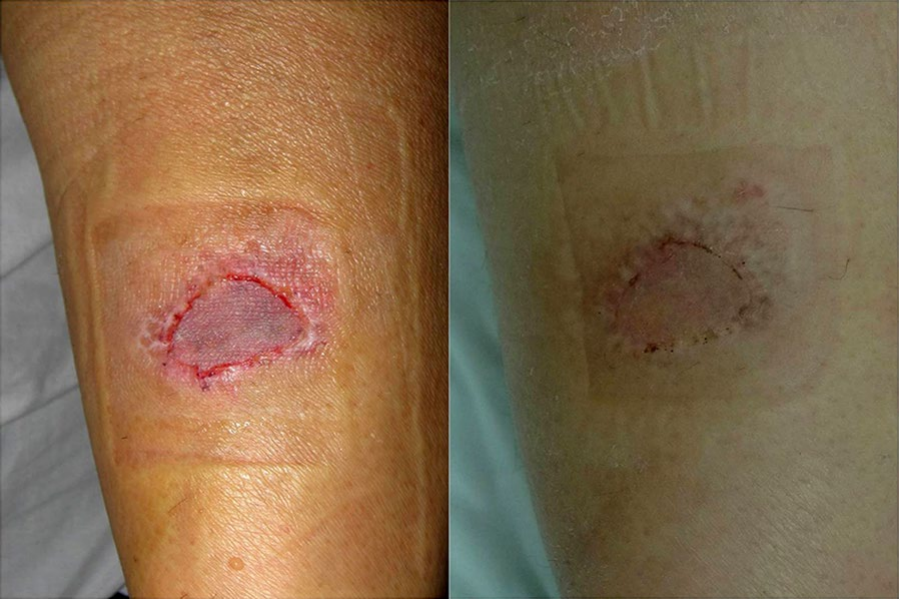
(*left*) Right leg contact burn in a 42-year-old man. No hematoma was seen 5 days after split-thickness skin grafting using fibrin glue. (*right*) No skin loss orsuture mark scar was observed 1.5 months postoperatively

**Fig. 3:**
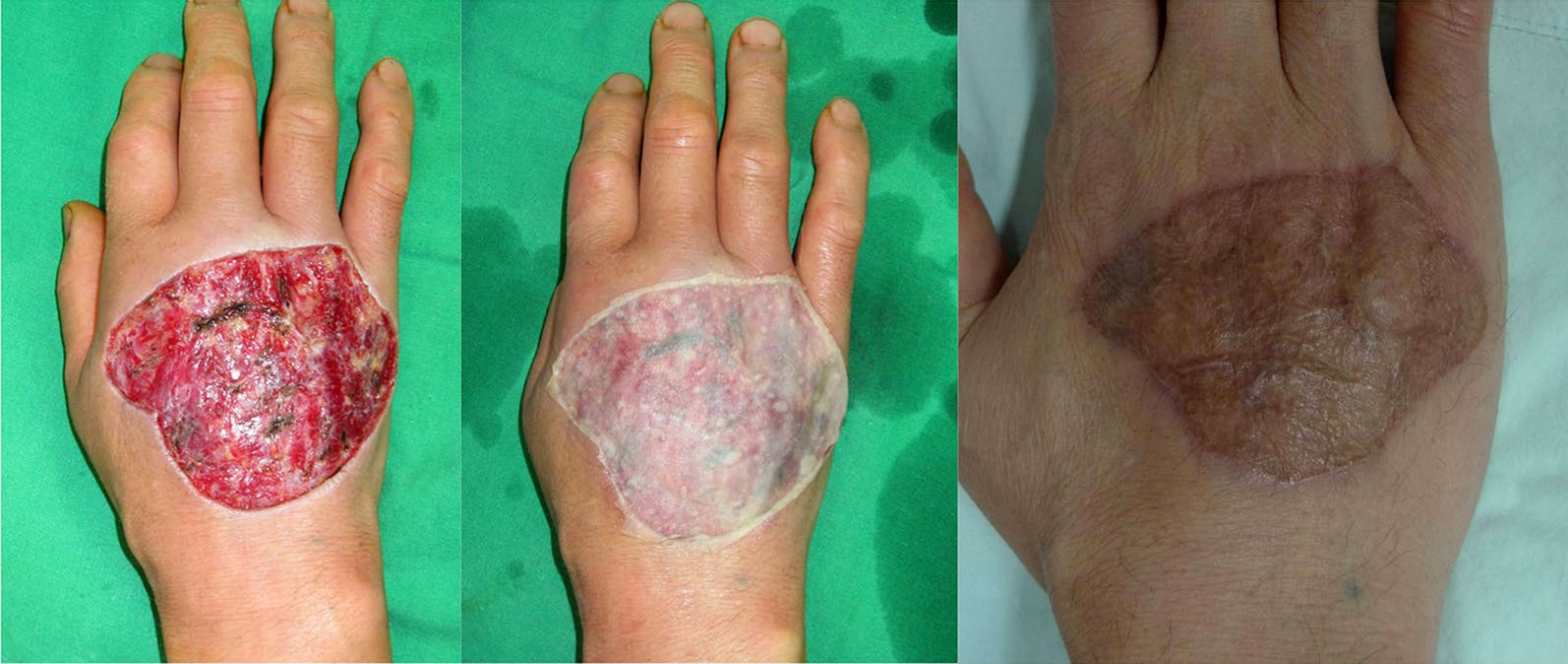
(*left*) Right hand friction burn in a 54-year-old man. (*center*) Split-thickness skin grafting was performed using fibrin glue. (*right*) No hypertrophic scar orsuture mark scar was observed 8 months postoperatively

Undiluted fast-clotting fibrin glue was used for graft fixation in 25 patients (group I). Greenplast (Green Cross Corp., Yongin, South Korea)—a fast-clotting fibrin sealant containing 400 IU/mL thrombin—was used in this study. During surgery, a marginal incision was made in the skin defect area, and an even layer of fibrin sealant was sprayed on the healthy wound. The skin graft was applied on the sealant layer, and fibrin sealant was applied once more to the graft boundary. Sutures and metallic staples were not used. Moderate compressive dressing was performed using mesh gauze and a gauze bandage (Figs. 1, 2, 3).

**Fig. 4:**
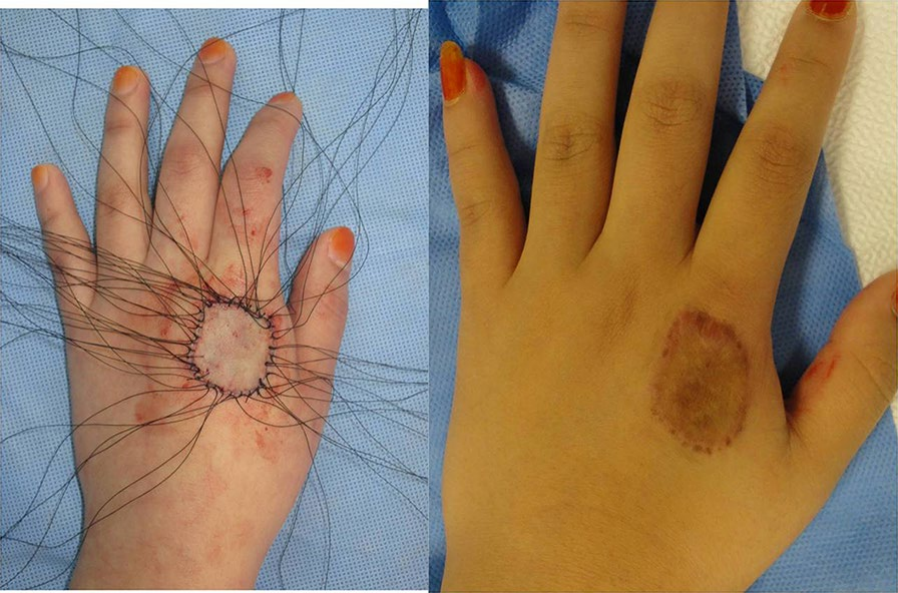
(*left*) Left hand contact burn in a 20-year-old woman. Split-thickness skin grafting was performed using sutures, and tie-over dressing was applied. (*right*) A hypertrophic scar and hyperpigmented suture scars were observed 6 months postoperatively

The classic graft fixation method using silk sutures or staples was performed in another 30 patients (group II). Fibrin glue was not used in this group (Fig. 4). Moderate compressive dressing same method with group I was also performed.

The dressing in the graft area was changed for the first time 5 days postoperatively, and hematoma/seroma formation and surface area of graft dislocation were investigated. At 30 days postoperatively, graft necrosis and graft take were investigated. Ratios of total surface area to hematoma/seroma formation, graft dislocation, graft necrosis, and graft take were obtained using Image J software (National Institutes of Health, Bethesda, MD, USA).

Statistical analysis was done using the independent *t* test and Pearson correlation coefficients. A value of *p* < 0.05 was considered statistically significant. Statistical analysis was performed using SPSS version 13.0 software (SPSS Inc., Chicago, IL, USA).

## Results

The major mechanisms of trauma in the 55 subjects were pedestrian and bike accidents and contact burns. Among the 55 subjects, all wounds were located on upper and lower extremities where the skin was hard to fix. The size of the defect area ranged from 3 cm × 2 cm to 22 cm × 5 cm. The median area of skin graft was 35.5 cm^2^ (the range 6–110 cm^2^) in group I versus 39.85 cm^2^ (the range 12–90 cm^2^) in group II. All subjects underwent split-thickness skin grafting, with a mean follow-up period of 16.4 months.

After 5 days, rates of hematoma/seroma formation and graft dislocation were 7.84 and 1.29 in group I, and 9.55 and 1.45% in group II, respectively. After 30 days, rates of graft necrosis and graft take were 1.86 and 98.14% in group I, and 4.65 and 95.35% in group II (Table 1). Undiluted fibrin sealant showed significantly superior results for all rates (*p* < 0.05) except graft dislocation and revealed superior outcomes compared with sutures except graft dislocation.

**Table 1:** Rates (%) of hematoma/seroma formation, graft dislocation, graft necrosis, and graft take

Fixation method	Hematoma or seroma formation at 5-days postoperative	Graft dislocation at 5-days postoperative	Graft necrosis at 30-days postoperative (the range)	Graft take (100 − necrosis rate) at 30-days postoperative (the range)
Fibrin glue with undiluted high-concentration thrombin (group I)	7.84	1.29	1.86 (1.06–4.5)	98.14 (95.5–98.94)
Silk sutures or staples (group II)	9.55	1.45	4.65 (2.23–6.65)	95.35 (93.35–97.77)
*p* value	<0.05	>0.05	<0.01	<0.05

**Fig. 5:**
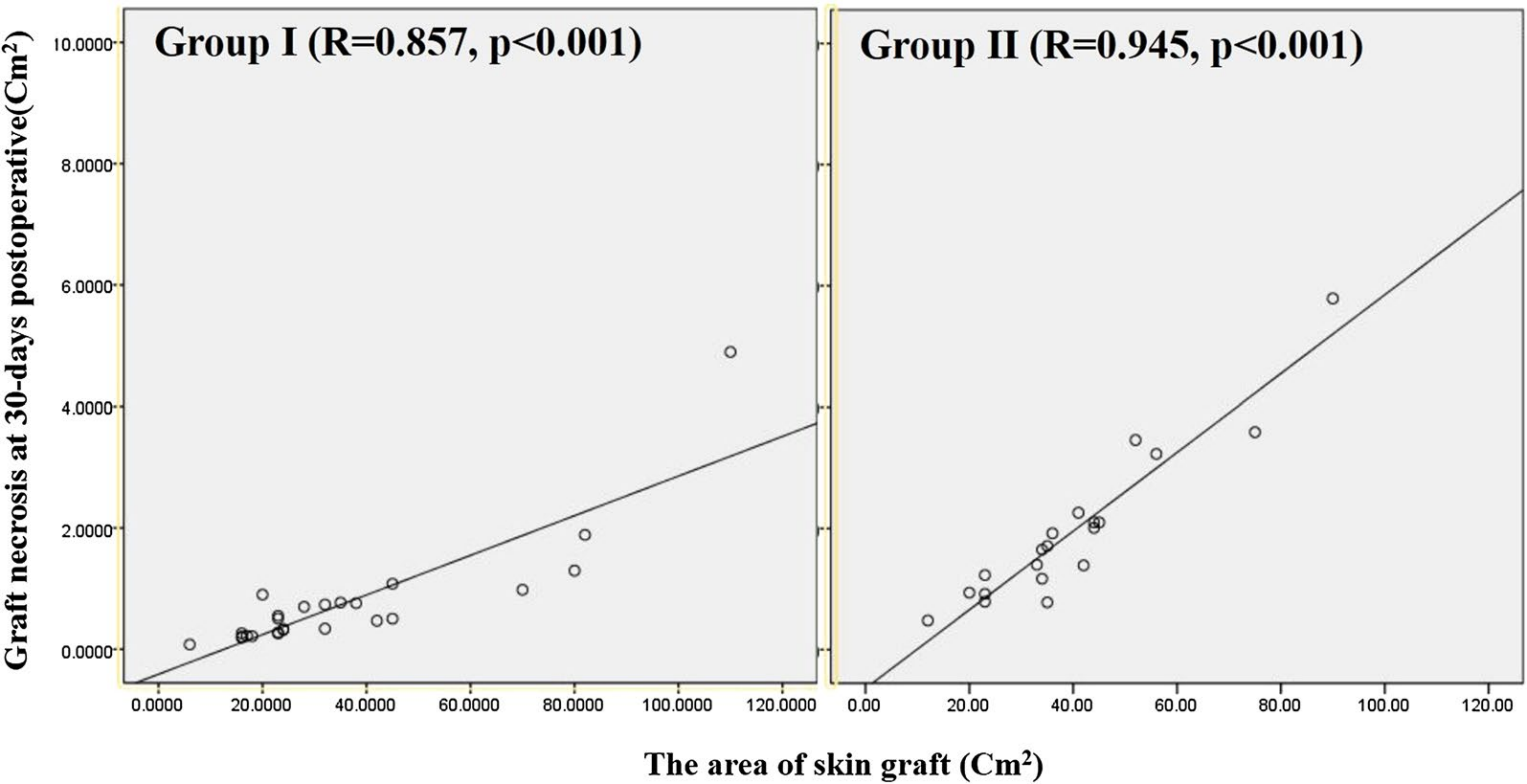
When analyzing the influence of the area of skin graft (cm^2^) and graft necrosis which means graft loss (cm^2^), we observed that in group II the area of necrosis is directly related to the size of the graft (R = 0.945, *p* < 0.001), while in group I this relation is also observed but showed the less stiffness of the slope (R = 0.852, *p* < 0.001)

When analyzing the influence of the area of skin graft (cm^2^) and graft necrosis which means graft loss (cm^2^) as the previous study (Llanos et al. 2006) showed (Fig. 5), we observed that in group II the area of necrosis is directly related to the size of the graft (R = 0.945, *p* < 0.001), while in group I this relation is also observed but showed the less stiffness of the slope (R = 0.852, *p* < 0.001).

## Discussion

Skin graft fixation using fibrin sealant has been widely performed since the late 2000s (Gibran et al. 2007; Mittermayr et al. 2006; Buckley et al. 1999; Currie et al. 2001; Llanos et al. 2006). Via hemostasis and adhesion, fibrin glue fixes the graft and creates an ultrafine fibrin sealant matrix structure to diffuse nutrients and cytokines, promote neovascularization, and facilitate growth of fibroblasts and capillary endothelial cells by serving as a scaffold. This structure then promotes phagocytosis and blocks the source of infection (Gibran et al. 2007; Mittermayr et al. 2006; Currie et al. 2001; Foster et al. 2008; Jabs et al. 1992).

Although skin graft fixation using fibrin sealant has proved useful for graft take, fibrin sealant is not widely used compared with sutures or skin staples, primarily due to difficulty in obtaining sealant containing low-concentration thrombin. Fibrin sealant contains thrombin and fibrinogen as active components; when these 2 ingredients are mixed, fibrin clots are formed (Sierra et al. 2000). When thrombin concentration is high, fibrin clots are formed quickly, whereas, when thrombin concentration is low, they are formed slowly. According to previous studies, low-concentration fibrin sealant containing approximately 4 IU/mL thrombin is recommended because fibrin clots are formed slowly, permitting graft placement. In addition, because such sealant is easy to manipulate, the graft take rate can be enhanced (Mittermayr et al. 2006; Grabosch et al. 1994). When undiluted fibrin sealant which contains a thrombin concentration of 400 IU/mL is applied to the human body, clots are formed quickly; thus, such sealant is used clinically as a hemostatic agent. Some fibrin sealants containing 4 IU/mL thrombin are restrictedly available in Europe. As an alternative, autologous blood can be collected to prepare the sealant, but the preparation process is complicated and time-consuming.

To overcome these problems, in this study, undiluted fibrin sealant was applied directly to the wound, and skin grafting was performed directly. Not only is the surgical method simplified, but the total time needed is shortened. The disadvantage of fast-clotting fibrin sealant is the formation of clots before the graft adheres to the wound bed, preventing their use as glue. When fast-clotting fibrin sealant was used in this study, clots were formed before graft application, and weaker adhesion was noted compared with low-concentration sealant. Nevertheless, little graft dislocation was observed at the time of first dressing change at 5 days postoperatively, and little graft necrosis was observed 30 days postoperatively.

Although small differences were found in graft take rate and graft necrosis between group I and group II, the numbers were statistically insignificant. It is common that many surgeons believe using staples and/or sutures are mandatory in graft fixation while using fibrin glue only makes them feel uncomfortable and insufficient. However, this study has demonstrated that using fibrin glue only in graft fixation is as effective as conventional methods. Furthermore, the results have even surpassed conventional methods particularly in joints and extremities.

For skin graft take, it is important to prevent hematoma/seroma development and the resulting inhibition of blood vessel ingrowth in the graft, which plays a critical role in graft failure. This study revealed that adhesion tension of the split-thickness skin graft during surgery was irrelevant to successful grafting. Fast-clotting fibrin sealant was proven to be effective for clinical use by avoiding hematoma formation.

In addition, the study has revealed that graft necrosis is proportional to area of skin graft both in groups. However, group I showed the less correlation coefficient compared to group II, it can be assumed that fibrin sealant (group I) could be more effective in large area grafting.

Sealant application thickness is also important for successful graft take. When the fibrin sealant layer is excessively thick, nutrition supply and neovascularization for the host and graft may be inhibited. This problem can occur with both fast- and slow-clotting fibrin sealants (Mittermayr et al. 2006). This problem can be solved by applying the sealant with a spray. A thin application with a thickness of 0.05–0.06 mL/cm^2^ has been reported to enhance the graft take rate (Mittermayr et al. 2006; Spies et al. 2001).

Use of fibrin sealant would be advantageous for patients if it could be obtained easily. Because fibrin sealant biodegrades within 2 weeks, foreign bodies, such as sutures and staples, are not left in the wound, especially in the granulation bed. In addition, patients often report severe pain when staples are removed, and occasionally, an anesthetic procedure is required (O’Grady et al. 2000; Batra et al. 2016; Himel et al. 1994; Best et al. 1995; Ghosh et al. 2015). Because fibrin sealant adheres to the entire surface, hematoma/seroma formation is significantly reduced compared with point-fixation sutures or staples (Cha et al. 2012; Gibran et al. 2007; Llanos et al. 2006; Myer et al. 2015; Mabrouk et al. 2013). According to previous studies, fibrin sealant is effective for graft take, particularly in joint and extremity areas that experience difficulty in postoperative immobilization. In addition, suture and staple marks are not formed, and the frequency of hypertrophic scar development caused by ischemic mechanical fixation is reduced (Mittermayr et al. 2006). Moreover, the enhanced graft take rate can result in decreased hospital stay as well as scar management costs, leading to overall cost savings even though fibrin sealant is more expensive and this cost could influence the total cost (Mittermayr et al. 2006). However, commercially available fibrin sealants contain bovine aprotinin and thrombin, which can theoretically result in transmission of Creutzfeldt-Jakob disease or other viral diseases (Currie et al. 2001).

As described previously, use of fibrin sealant can overcome the disadvantages associated with sutures and staples. Fibrin sealant has been used for skin graft fixation since 2000, and comparative studies on the thickness of the sealant layer have been reported (Mittermayr et al. 2006; Spies et al. 2001). However, few studies on the effect of thrombin concentration have been published. A limitation of this study is lack of comparison between low- and high-thrombin concentration groups. And randomized trials will need to be performed in order to verify our result.

## Conclusion

Although fibrin sealant has proved useful for skin graft fixation and graft take, it is not widely used compared with sutures or skin staples. When high-concentration fast-clotting fibrin sealant containing a thrombin concentration of 400 IU/mL was applied to skin grafts without dilution, no difficulty was experienced during surgery. Sealant showed superior results compared with sutures, and had an excellent graft take rate.
